# Protein over-expression in *Escherichia coli* triggers adaptation analogous to antimicrobial resistance

**DOI:** 10.1186/s12934-020-01462-6

**Published:** 2021-01-11

**Authors:** Jack James, Benjamin Yarnall, Andy Koranteng, Jane Gibson, Tahmina Rahman, Declan A. Doyle

**Affiliations:** 1 School of Biological Sciences, University of Southampton, Highfield Campus, Southampton, SO17 1BJ UK; 2 Cancer Sciences, Faculty of Medicine, University of Southampton, Southampton, SO16 6YD UK; 3 Queen Alexandra Hospital, Portsmouth Hospital University NHS Trust, Cosham, Portsmouth, PO6 3LY UK

**Keywords:** *Escherichia coli*, Antimicrobial resistance, BL21(DE3), Protein over-expression

## Abstract

**Background:**

The *E. coli* pET system is the most widely used protein over-expression system worldwide. It relies on the assumption that all cells produce target protein and it is generally believed that integral membrane protein (IMP) over-expression is more toxic than their soluble counterparts.

**Results:**

Using GFP-tagged proteins, high level over-expression of either soluble or IMP targets results in > 99.9% cell loss with survival rate of only < 0.03%. Selective pressure generates three phenotypes: large green, large white and small colony variants. As a result, in overnight cultures, ~ 50% of the overall cell mass produces no protein. Genome sequencing of the phenotypes revealed genomic mutations that causes either the loss of T7 RNAP activity or its transcriptional downregulation. The over-expression process is bactericidal and is observed for both soluble and membrane proteins.

**Conclusions:**

We demonstrate that it is the act of high-level over-expression of exogenous proteins in *E. coli* that sets in motion a chain of events leading to > 99.9% cell death. These results redefine our understanding of protein over-production and link it to the adaptive survival response seen in the development of antimicrobial resistance.

## Background

The *Escherichia coli* pET system [1] uses a genome encoded T7 RNA polymerase (T7 RNAP) to control the production of the target mRNA (Additional file 1: Fig. S1). The pET system is extensively used in biotechnology, pharmaceutical and scientific research fields involving the production of biofuels [2], bioproducts [3], metabolites [4] genetic research [5], long-term evolution studies [6], multi-omics research [7] and metabolic engineering [8]. For successful exogenous protein production, the T7 RNAP and the recombinant target protein is regulated via the addition of isopropyl β-D-1-thiogalactopyranoside (IPTG). The recommended IPTG concentration ranges from 0.4 to 1 mM [1]. For over 30 years since its introduction [9], the fundamental principle for this process is that, after IPTG addition, all cells produce T7 RNAP and the target mRNAs. In order to increase the production of more target mRNA, and thus more target protein, trial and error screening of protein production-based variables are required due to our lack of understanding of the system such as altering culture growth temperature, varying the length of time of protein over-expression, changes in culture medium, changing inducer concentration, the use of additives for example glycerol, specific ions or even changing the expression system.

Protein structural studies require µg to mg of purified protein for structural determination. The pET system is the most widely used method of protein production in *E. coli *[10, 11]. Over-production of integral membrane proteins (IMPs) has proved problematic due to low expression levels, folding problems, lack of activity after over-expression and potential toxicity to cells. To overcome these issues, mutant strains have been developed including C41(DE3) and C43(DE3) [12]. The effectiveness of the C41(DE3) strain was based on the lacUV5 promoter site mutations that drops the expression level of the T7 RNAP mRNA down to that of the weaker lac wildtype promoter [13–15]. C43(DE3) also had a mutation in the lacI IPTG binding site that lowered its affinity for IPTG thus reducing protein induction levels [14, 15]. C44(DE3) and C45(DE3) mutants contain stop codons within the T7 RNAP gene [16] which are naturally overridden. This process occurs infrequently hence these mutants produce full length T7 RNAP mRNA but at a reduced level as compared with BL21(DE3). Mutant56(DE3) was found to have a T7 RNAP mutation that lowered the affinity of the polymerase for its T7 promoter site [17]. Introduction of a mutation into the sigma 70 factor (rpoD-E575V) of BL21 decreased the affinity for the endogenous RNA polymerase thus leading to down regulation of the target IMP mRNA [18]. Another approach involves controlling the activity T7 RNAP using titratable amounts of the inhibitor, T7 lysozyme, as implemented in the Lemo21(DE3) strain [13, 19]. An alternative method of controlling T7 RNAP activity is to co-express either the pLysS or pLysE plasmids. These plasmids allow for the constitutive expression of T7 lysozyme which is a natural inhibitor of the polymerase. Therefore, by inhibiting the polymerase less target protein is generated. In addition, the levels of T7 RNAP mRNA have been controlled using a mutant lacI repressor that is less responsive to IPTG [20]. Likewise, the BL21-AI strain was developed to tightly regulate the expression of T7 RNAP and was used to successfully overexpress a ‘toxic’ viral ion channel [21]. These mutants and modified strains were developed for the over-expression of IMPs in E. coli, with the goal of decreasing the amount of recombinant IMP mRNA via lowering of the polymerase mRNA levels. It has been assumed that this approach works because over-expression of IMPs is generally believed to be toxic thus decreasing IMP expression levels allows *E. coli* to survive.

We investigated the mechanism of protein over-expression using superfolder Green Fluorescent Protein [22] (sfGFP)-tagged versions of both soluble and membrane proteins. These were expressed under varying IPTG concentrations and colony forming units (CFUs) were counted. This allowed characterisation of expression levels of all target proteins and correlated the CFUs with their phenotypes. Our data demonstrates that it is the act of over-expression of all of our target proteins, and not just integral membrane proteins, that is toxic to *E. coli*. In response to this toxicity, *E. coli* undergoes selective pressure producing an adaptive selective response that utilises mutant strains to decrease or remove T7 RNAP expression.

## Results and discussion

### Three surviving phenotypes after the loss of > 99.9% of viable cell culture

**Fig. 1:**
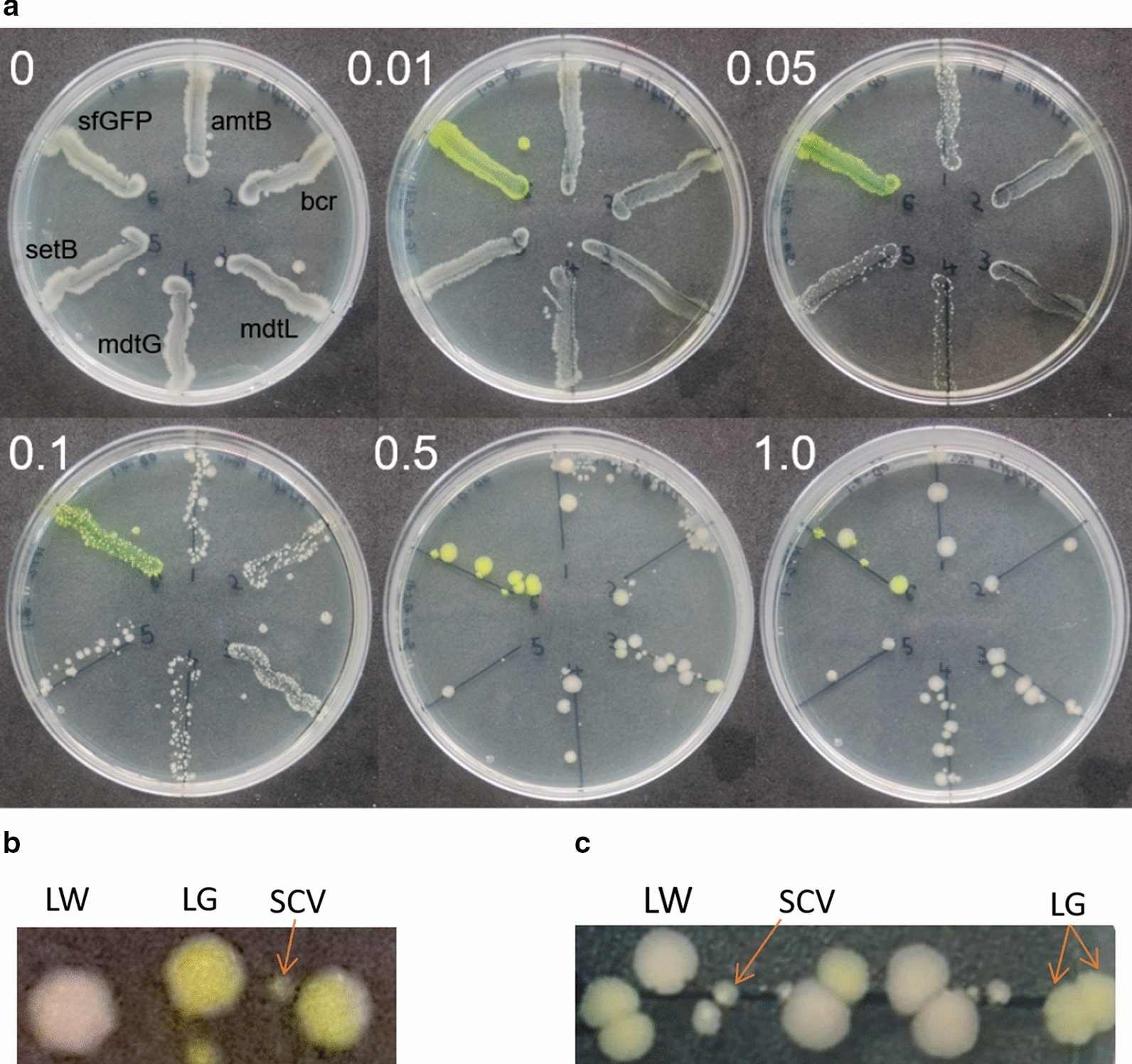
Streak plating to show the effect of increasing IPTG concentration on CFU formation.** a** Protein over-expression in BL21(DE3) and CFU numbers for five IMPs and sfGFP. The final IPTG concentration (mM) is indicated. All cultures were plated at OD_600_ of 0.1. **b** Close up view of sfGFP sample at 1 mM IPTG showing 1 LW, 1 LG and 4 SCVs. **c** Close up view of mdtL-sfGFP at 0.5 mM IPTG showing 4 LW, 5 LG and 5 SCVs

To test if all transformed BL21(DE3) cells over-express target protein (Additional file 1: Fig. S1), *E. coli* was streaked onto LB agar plates with increasing IPTG concentration (Fig. 1a). A variety of sfGFP-tagged IMPs along with soluble sfGFP were streaked for comparison including an ammonium transporter (amtB) [23], three drug transporters (bcr [24], mdtL [25], mdtG [25]), a sugar transporter (setB [26]) and the soluble protein sfGFP [22]. No exogenous overexpressed protein was observed at 0 mM IPTG. The expression of sfGFP is clearly visible throughout and, as anticipated, IMPs expression is significantly lower. Against expectation, as the IPTG concentration increased to 1 mM, the number of CFU counts dramatically fell from a confluent growth to only two CFUs in some cases (Fig. 1a; setB-sfGFP). Phenotypically, the CFUs formed three categories described as large and green (LG), large and white (LW) or small colony variants (SCVs; Fig. 1b, c, Additional file 1: Fig. S2). This applied to both IMPs and the soluble protein.

**Fig. 2:**
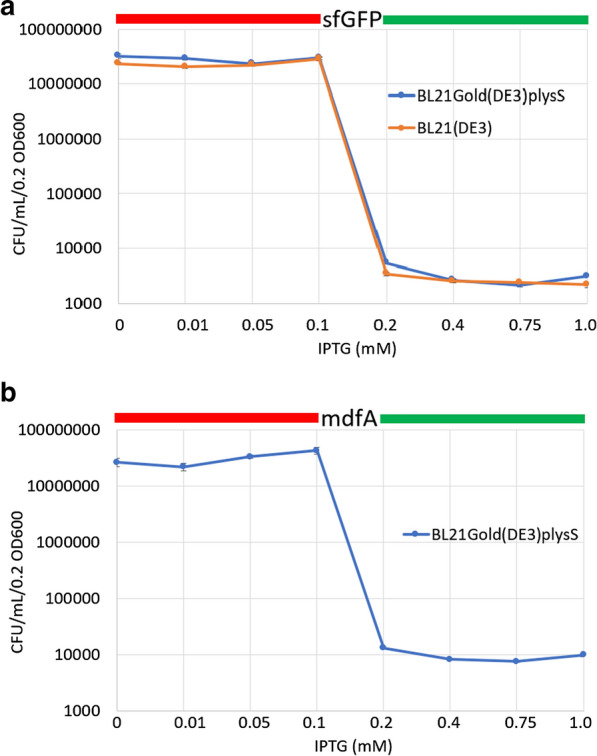
Change in CFU numbers with increasing IPTG concentration. For all IPTG concentrations, the same cell culture density was used. **a** *E. coli* BL21-Gold(DE3)pLysS and BL21(DE3) strains were transformed with the soluble protein sfGFP while, **b** BL21-Gold(DE3)pLysS was transformed with the integral membrane protein mdfA. For both figures, standard error bars are included (for **a** n = 8 to 12 while for **b** n = 5–6)

Next, these results were quantified using the well characterised protein targets sfGFP and mdfA [27, 28]. Both proteins were chosen because they have been overexpressed to milligram quantities sufficient for structure determination by X-ray crystallography [29, 30]. Two *E. coli* protein production strains, BL21(DE3) and BL21-Gold(DE3)pLysS (Agilent Technologies), were chosen. Surprisingly, both the soluble protein (Fig. 2a) and the IMP (Fig. 2b) behave identically with the CFU numbers remaining high from 0 to 0.1 mM IPTG concentrations followed by a collapse in numbers at IPTG concentration above 0.1 mM. In addition, both *E. coli* strains behaved identically (Fig. 2) suggesting that the strain does not account for this sharp drop in CFUs. Calculating the percentage drop for the soluble sfGFP protein, by averaging the CFU count between the 0–0.1 mM IPTG counts (Fig. 2a, red bar; 26.19 million) and averaging between 0.2–1.0 mM IPTG counts (Fig. 2a, green bar; 3000 CFUs) gives a population decrease of 99.99%. Similarly, for the IMP mdfA the 0–0.1 IPTG counts averaged 31.42 million CFUs (Fig. 2b, red bar) while the 0.2–1.0 mM IPTG CFUs averaged 9713 (Fig. 2b, green bar) which equates to a fall of 99.97%. Hence, for both IMP and the soluble targets, exogenous protein production can only come from < 0.03% of the initial total number of bacteria.

Theoretically, when using > 0.1 mM IPTG, high expression levels of the T7 RNAP (Additional file 1: Fig. S1) could result in the observed ‘toxic’ effect (Fig. 2). To test this, fresh, untransformed BL21(DE3) cells (no expression vector present) were plated at an OD600 of 0.2 on LB agar plates containing 0 and 0.4 mM IPTG and CFUs were counted. If the overproduction of T7 RNAP is causing the toxic effect, then we would expect 3–9 thousand CFUs at 0.4 mM IPTG. To the contrary, both IPTG concentrations resulted in high counts (0 mM IPTG: 29.3 ± 3.0 million (n = 6) and 0.4 mM IPTG 36.6 ± 5.0 million (n = 6)). This confirmed Schlegal et al. [15] data hence toxicity is not due to T7 RNAP. Therefore, is the fall in CFUs because of the presence of the vector? This can be determined from Fig. 2. The average combined CFU counts at 0 mM IPTG (expression vector present but no protein induction) for both *E. coli* expression strains of 27.1 ± 8.4 million (n = 25) is at the same previous high level in the absence of vector. Therefore, we can exclude IPTG, the presence of uninduced vector, the type of protein (IMP or soluble), the *E. coli* strain or T7 RNAP as the cause of the toxic effect.

### Genomic mutations are central to the *E. coli* response

To determine the potential genetic changes as a result of protein over-expression, the genomes of six samples (1–6) and two controls (7–8) were sequenced (Additional file 1: Table S1). Samples 1–4 were taken from single colonies that were exposed to 1.0 mM IPTG. Samples 5 and 6 were produced by exposing cells to only 0.01 mM IPTG. This low level of IPTG was not expected to be a metabolic burden to the cells based on the CFU counts (Fig. 2). This was confirmed by the generation of a lawn of visible green cells for both types of proteins (Additional file 1: Table S1). We assumed therefore that at 0.01 mM IPTG all cells behaved identically therefore many cells, as part of a swipe, were used as the sample for genome sequencing. Samples 7 and 8 contained the expression vectors but were never exposed to IPTG.

Genomic sequencing of control samples 7 and 8 indicated that there were three missense background mutations, not related to protein expression. (Additional file 1: Table S2). The genome sequencing results confirm that, if uninduced, the expression vector causes no major genetic changes.

In comparison with the BL21-Gold(DE3)pLysS published genome template, there were eleven mutations of which nine were common to samples 1–6. These six samples were generated using two different IPTG concentrations and two vectors hence the common mutations can be considered as background mutations already present in our laboratory strain. The remaining two genetic changes were linked to the observed LW and LG phenotypes.

**Fig. 3:**
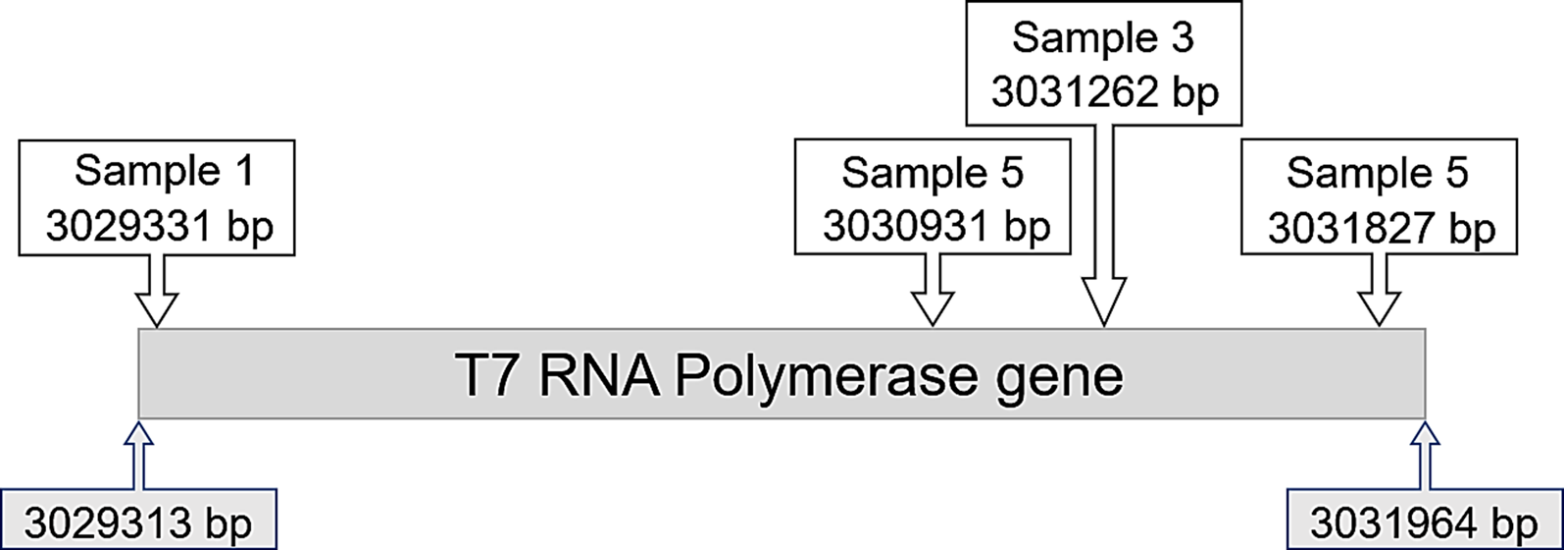
A schematic representation of the IS10 insertion sites in the genome encoded T7 RNAP gene. The start and ending base pair numbers are indicated below the bar while the location of the IS10 insertions are indicated above. Base pair locations relative to reference sequence: gi|253322479|gb|CP001665.1| E. coli BL21-Gold(DE3)pLysS AG

The lack of visible green colour from the LW colonies implies that they are not synthesising target protein. The sequencing results confirmed this assumption as, for both the soluble and membrane proteins, the T7 RNAP has been disrupted by a single 1293 base pair IS10 insertion within the gene (Fig. 3). IS10 is the transposase of the Tn10 transposon. These mutant T7 RNAP’s activity were not measured but the known properties of IS element integration [31–33] and the lack of colour indicate a non-functional enzyme.

The percentage of LW CFUs differs between BL21(DE3) and BL21-Gold(DE3)pLysS (Additional file 1: Table S3). Higher LW CFU numbers for BL21-Gold(DE3)pLysS may be due to the presence of the IS10 in its genome. The BL21(DE3) genome does not carry the IS10 insertion sequence [34, 35]. A possible reason for BL21(DE3)’s LW phenotype could be explained by C44(DE3) and C45(DE3) strains which have stop codons within the T7 RNAP gene [16].

For sfGFP and mdfA-sfGFP, LG mutations were identical. As previously observed in C41(DE3) the mutation resulted in the conversion of the lacUV5 promoter’s -10 site (TATAAT) back to the lacI-10 sequence (TATGTT). This change is known to result in a tenfold lower T7 RNAP transcriptional rate [12, 13, 15]. Surprisingly, the same mutation was also found in ~ 10% of the multi-genome Sample 5 reads. Sample 5 was generated from sfGFP over-expression in BL21-Gold(DE3)pLysS using 0.01 mM IPTG. The fact that identical mutations are found in the LG colonies for both the soluble protein and the membrane protein as well as in the sample exposed to only 10 µM IPTG is consistent with the phenomenon of adaptive mutation i.e., non-toxic selection produces mutations that relieve the selection pressure [36]. This is supported by the presence of two additional T7 RNAP IS10 insertions identified in a 2% subset of Sample 5 (Additional file 1: Table S2, Fig. 3). These two additional sites are also likely to render the T7 RNAP inactive.

## High-level exogenous protein over-expression is bactericidal

As we have shown, induction of exogenous protein results in very large cell losses and a survival rate of < 0.03%. Is the process bactericidal or bacteriostatic? Replica plating lawns of cells grown with 0.75 mM IPTG onto LB agar with no IPTG demonstrated that the process was bactericidal (Additional file 1: Fig. S3). Hence, from Fig. 2 the minimal bactericidal concentration for protein over-expression is 0.2 mM IPTG. To clarify this point, IPTG is not an antibiotic but the bactericidal effect is only seen when IPTG is used for high level exogenous protein over-expression in combination with T7 RNAP and the expression vector containing an in-frame coding gene.

### In liquid culture, ~ 50% of all cells produce no exogenous target protein

**Fig. 4:**
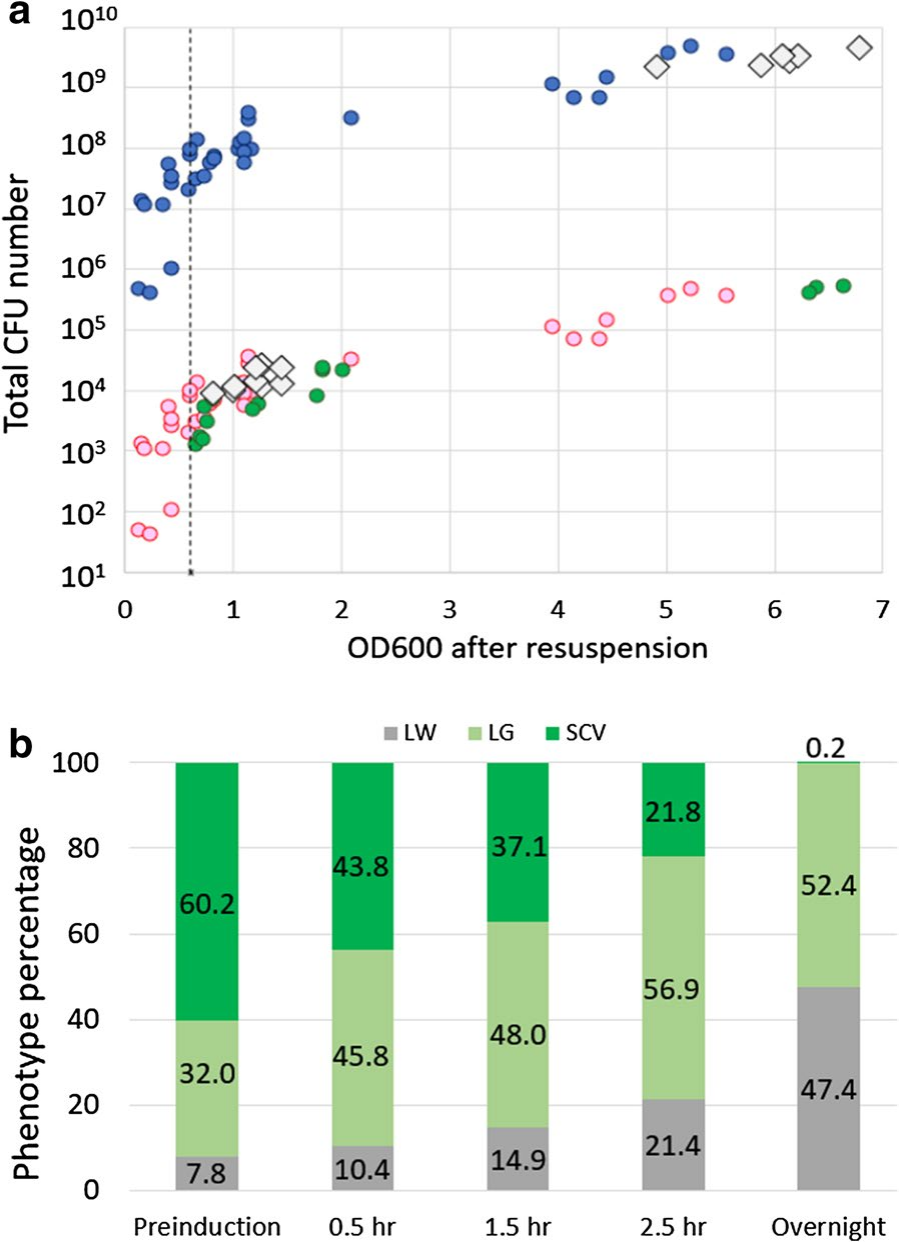
Effect of protein over-expression on the phenotype population in solution upon protein induction with 0.4 mM IPTG. **a** The change in total CFU number (LW + LG + SCV) with increasing OD600. Values above OD600 > 3.5 are overnight samples. Blue circles indicate the CFU numbers from BL21(DE3) transformed with H6msfGFP and plated on LB agar plus kanamycin. The red circles are the predicted 99.99% decrease in CFU numbers (Fig. 2) as a result of the induction of exogenous protein over-expression. The green spheres are the measured CFU counts due to exogenous protein over-expression induction on LB agar plates only. Clear diamonds are the measured CFU numbers due to exogenous protein over-expression (protein induction) when 0.4 mM IPTG is added to the liquid medium. The dashed line represents the recommended protein induction OD600 value of 0.6. All measured points are an average of duplicate plating. **b** Percentage of LW, LG and SCV phenotypes observed during the protein over-expression experiment. Preinduction samples were taken at an average OD600 of 0.5 (Additional file 1: Fig. S4b). Protein over-expression was started approximately seven minutes later with the addition of 0.4 mM IPTG. The remaining samples were taken at the indicated times after the start of protein over-expression. Overnight represented ~ 14.5 h after the start of exogenous protein over-expression. The phenotype percentages for each of the sampled points were generated from CFU counts based on six individual cell cultures and the averaging of duplicate plates for each individual point

Finally, we examined what happens in solution because this is the usual method of protein over-production. Using the recommended protocol [1], BL21(DE3) cells transformed with the sfGFP vector were grown in LB. Samples were taken before and after the induction of exogeneous protein over-expression and plated on the appropriate LB agar plates (Fig. 4 and Additional file 1: Fig. S4). Phenotype and CFU numbers were counted. As expected, uninduced control samples produce CFU counts > 10^6^ per mL with higher OD600 values producing higher CFUs (Fig. 4a; blue circles). Plating these cells on LB agar + 0.4 mM IPTG should result in the 99.99% decrease due to the bactericidal effect. These predictions are plotted as red circles in Fig. 4a. The measured CFU counts, when plated on 0.4 mM IPTG plates (green circles), confirm this prediction. Next, exposure of the cells to 0.4 mM IPTG in the liquid medium begins the mutant (LG, LW, SCV) selection process. Therefore, we expect that non-mutant cells would die, and, in time, the mutant phenotypes would grow to numbers equivalent to WT levels (Additional file 1: Fig. S4). This is indeed observed since, in overnight cultures, the total number of CFUs reaches the trillions (Fig. 4, open diamonds) in line with the observed uninduced results (blue circles). The difference between these two groups is confirmed in the final phenotypes as WT cultures (blue circles) were always uniform in size and colourless (Additional file 1: Fig. S2 at 0 mM IPTG) while the IPTG exposed cultures were LG, LW and SCV variants.

Quantification of the various phenotypes throughout the over-expression trial showed a re-distribution of the proportion of mutants over time (Fig. 4b). There is a clear gradual dominance in the LG and LW mutants, eventually resulting in an almost 50:50 LG:LW ratio in overnight cultures.

It is believed that *E. coli* over-expression of some exogenous target proteins can be toxic, particularly so for IMPs [12, 37]. In addition, it is often found that reliable expression protocols suddenly fail, or protein expression levels vary from prep-to-prep. Our data shows that the manufacturer’s 0.4 mM IPTG recommendation [1] results in selection of the LW, LG and SCV phenotypes. Larger LG and LW CFUs compared with SCV imply a faster doubling rate. Therefore, by the end of a protein over-expression experiment, LW and LG numbers dominate (Fig. 4b). Even though the average overnight LW proportion was ~ 50%, the measured values ranged from 30–70%. The initial LW population also varied from ~ 3–16% (Additional file 1: Table S3). This significant variability in total amount of non-productive LW cells would contribute to the observed prep-by-prep inconsistencies. Our work points to the variation in LW, LG and SCV proportions rather than clonal instability [15] as the cause of variability and provides an explanation for Miroux and Walker (1996) [12] data in which over-expression of the F-ATPase subunit b decreased over an extended period of time due to ‘lost expression capacity’.

## Conclusions

This study used five IMPs and one soluble protein. Published images of BL21(DE3) over-expressing proteins confirm our results: Schlegel et al. [15] examined four soluble proteins and three IMPs, while Miroux and Walker [12] looked at two IMPs (Additional file 1: Table S3). They all produce the combination of large CFUs and SCV strains when using > 0.1 mM IPTG. In addition, the use of different vectors and antibiotic selective markers allows their exclusion as the cause of toxicity. As we have excluded a single entity as the cause, we therefore propose that it is the act of over-expressing *any* exogenous target protein at high levels that is toxic. This would produce excessive amounts of exogenous mRNA that outcompete endogenous mRNA for ribosomes [38–40]. Thus, endogenous cellular proteins cannot be synthesised in sufficient quantities for cell viability.

When finding the optimum conditions to maximise target protein production a trial and error approach using two time periods (2–4 h or overnight) is used. Our results help to explain why two separate time frames are employed. There are significantly more SCV cells in the short 2.5 h induction time as compared with overnight expression (Fig. 4b). As the production capacity and the physical numbers of the LG and SCV variants are expected to produce varying amounts of target protein, the SCVs are likely to play a role in protein over-expression but only for short induction protocols. Importantly, for overnight expression trials, on average the 50% LW cells make no exogenous protein. Consequently, new expression protocols or additional strains may be required to optimise protein over-expression. These should aim to prevent the unproductive growth of LW cells while boosting exogenous protein production via LG, SCV or related strains.

**Fig. 5:**
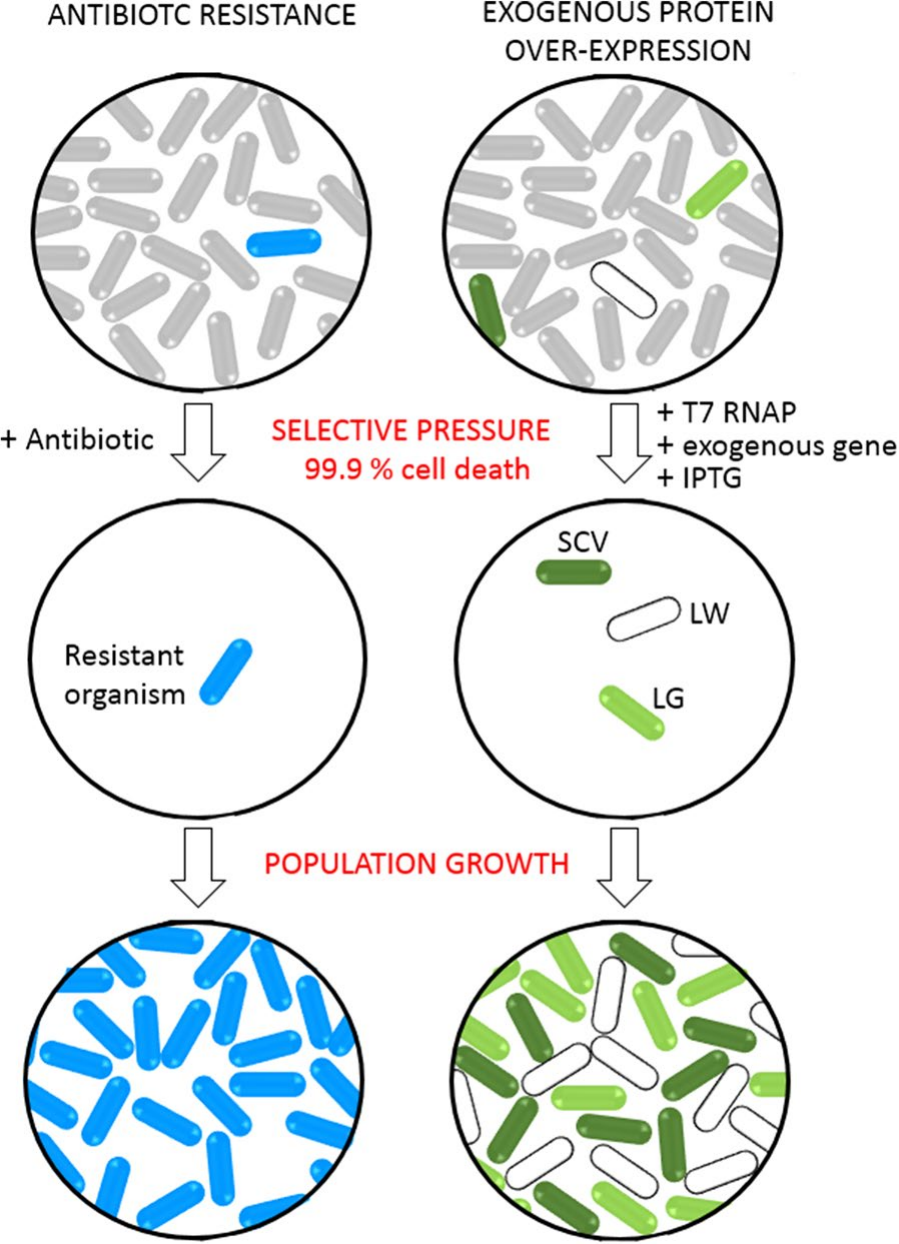
Comparison of the processes of the development of bacterial antibiotic resistance with exogenous protein over-expression in *E. coli*. Non-resistant cells and BL21(DE3) bacterial cells are both shown in grey. Exogenous protein production will only occur in the LG and SCV variants

The cellular response to a bactericidal antibiotic is > 99.9% drop in viable bacteria. This attack produces a selective pressure resulting in the development of antibiotic resistance (Fig. 5). Additional characteristics include the selection of mutants and SCVs [41, 42]. Our results show a bactericidal population drop of > 99.9% (Figs. 2,  4, Additional file 1: Fig. S3). Survivors contain mutations in the T7 RNAP (Fig. 3) or the lacUV5 promoter (Additional file 1: Table S3) and SCV formation is observed (Fig. 1, Additional file 1: Fig. S2). Similar findings of slow growth and SCV formation are known to occur when cells are stressed e.g., during antibiotic exposure [41, 42]. Tashiro et al. [43] demonstrated that a slow growing, antibiotic resistant *E. coli* SCV was formed from Tn1000 transposon gene insertion while a mutation in the yigP gene generated *E. coli* SCVs [44] resulting in antibiotic resistance.

Based on our results, the terms ‘toxic’ or ‘toxicity’ that are extensively used to describe some proteins during over-expression experiments, are incorrect. Most of these proteins are in fact not toxic as they have been over-expressed to high levels using C41(DE3) strain which functionally only has the lacUV5 to lacI promoter mutation i.e., no other major biochemical or genetic changes occur [11, 12, 15]. Thus, many of the so-called ‘toxic’ proteins are harmless. This misconception stems from historical protocols which have focussed on soluble proteins. The situation is different for membrane proteins. The over-expression of integral membrane proteins which, due to their nature, their final cellular destination, their folding and insertion processes, automatically means that expression levels are generally much lower than for soluble proteins. Thus, membrane protein over-expression has been tackled after their soluble counterparts. Therefore, the initial over-expression protocols were poorly optimised for membrane proteins.

The underlying protein over-expression mechanism is similar to the development of antimicrobial resistance based on our demonstration of the following six observations: (1) the presence of a selective pressure (Fig. 5 and main text), (2) the drop in CFU population by > 99.9% (Fig. 2), (3) demonstrating that it is bactericidal (Fig. 1, Additional file 1: Fig. S3), (4) the process is concentration dependent (Fig. 2), (5) it involves the generation of mutants that are resistant (genome sequencing Additional file 1: Tables S1 and S2) and (6) low IPTG concentrations preparing cells for an attack as do antibiotics at concentrations below their minimal bactericidal/inhibitory concentrations (Additional file 1: Tables S1 and S3 and main text). However, in contrast to antibiotics, IPTG is not toxic to *E. coli*. In this case, the selective pressure is the process of high-level protein over-expression. It is this pressure that generates the three phenotypes LG, LW and SCV. This data shows, for the first time, how each phenotype relates to exogenous protein over-expression (Fig. 4).

In conclusion, all biological experiments ultilising the pET system, will need to consider the presence of genomic mutations driven by the protein over-expression process. We suggest that this can be overcome by genome sequencing verification of the production strain, using alternative expression strains such as HMS174(DE3) [45], using the weaker inducer lactose [46, 47], controlled-down regulation of T7 RNAP production [19, 20], making use of alternative mutant expression strains, using a cell-free system [48] or a combination of new strains and weaker induction. In light of our new results, we suggest two possible adaptations to the existing protein over-expression protocols with the aim of reducing the total number of unproductive LW phenotypes. Firstly, using an IPTG concentration of < 0.1 mM IPTG (Fig. 2) will prevent the dramatic loss of total cell population hence almost all cells will be able to produce the target protein. Secondly, using > 0.1 mM IPTG will result in LW, LG and SCV. However, limiting the total over-expression time to 4–8 h (Fig. 4) will lower the total LW numbers therefore boosting numbers of the productive LG and SCV phenotypes.

## Methods

### *E. coli* K12 MG1655 genomic DNA preparation

The integral membrane protein genes were cloned using *E. coli* K12 MG1655. The template genomic DNA was purified using a modified procedures and buffers from a Fermentas GeneJET Plasmid Miniprep kit (Cat. No. K0502). From a 3 mL LB overnight culture of *E. coli* K12 MG1655 grown at 37 °C and 180 rpm, the cell pellet was resuspended, lysed and then the solution neutralised as per the manual instructions. At this stage, the sample was vigorously shaken using a vortex for 20 secs. Bench top centrifugation at maximum speed for 3 min allowed for the separation of cell wall and precipitated materials, leaving the fragmented genomic DNA in solution. To the ~ 800 µL of supernatant, 600 µL of room temperature isopropanol was added and mixed gently to precipitate the DNA. After centrifugation at 15,000 *g* for 2 min the supernatant was carefully removed before being washed with 600 µL of 70% ethanol. The centrifugation step was repeated, and all supernatant carefully removed before allowing to air dry for 10–15 min. Once dry, rehydrate with 100 µL of Tris/EDTA buffer by incubating for 1 h at 65 °C. Intermittently gently flick the tube to aid dissolving of the DNA pellet. Solve in − 20 °C freezer until use.

### Ligation independent cloning of genes into H6msdGFP

Lyophilised primers (Additional file 1: Table S4) ordered from Sigma-Aldrich (0.025 OD scale) were made to 100 µM using deionised water. For the 50 µL PCR reactions, the final concentrations for the following components were used: Primers 4 µM, dNTPs 0.4 mM and 8 pg/mL of template DNA. 0.5 µL of Phusion HF polymerase (Thermo Fisher Scientific, catalogue number F-518) and the recommended manufacturer’s buffer was used. The PCR protocol involved 30 repeat cycles of 98 °C denaturation for 30 secs, 55–60 °C annealing for 30 s and 72 °C extension for 60 secs after which an additional 5 min at 72 °C was given. After running the PCR samples in a 1% agarose gel in TAE buffer (40 mM Tris, 20 mM acetic acid, 1 mM EDTA), the bands of the expected size were excised using a clean blade. The DNA fragments were purified using the Thermo Scientific GeneJET Gel Extraction Kit. Before the generation of overhangs, H6msfGFP (Addgene; plasmid number 29725) was linearised with SspI (Thermo Fisher Scientific). After a cleaning step (GeneJet DNA Purification kit), the cleaved vector was treated with T4 polymerase in the presence of 2.5 mM dGTP. The purified PCR samples were also treated with T4 polymerase except in the presence of 2.5 mM dCTP. Following T4 treatment, samples were incubated at 75 °C for 20 min before purifying the DNA using the GeneJet DNA Purification kit. Before transformation into MACH1 commercially competent *E. coli* cells (Thermo Fisher Scientific), the T4 polymerase-treated vector and insert were incubated together at a 1 vector:4 insert ratio for 30 min at RT. All vectors with the correct inserts were confirmed by sequencing (Eurofins, Germany).

### Streak plating method

Freshly transformed BL21(DE3) cells with the vector containing the target genes were grown in 30 mL of LB containing 50 µg/mL kanamycin, at 37 °C and 180 rpm until reaching an OD600 of between 0.1 to 0.2. After centrifuging 5 mLs of cell culture to remove the supernatant, cells were washed twice in ice cold Resuspension buffer (0.1 M Tris pH 7.5, 0.1 M NaCl), via consecutive cycles of resuspension and centrifugation. The cell pellet was resuspended in 1 mL of ice-cold Resuspension buffer before adjusting the final OD600 to 0.1. An inoculation loop was used to streak the culture onto LB agar plates containing 50 µg/mL kanamycin in one step. The loop was flamed twice between culture samples. Plates were tightly sealed using parafilm before leaving them in a 37 °C incubator for between 12 and 72 h. The extended 72 h was used to allow to produce the GFP-tagged membrane proteins to reach levels that could be seen by eye. No additional colony forming units of any phenotype appeared after the initial 12 h of incubation.

### Cell storage, Resuspension OD600 and CFU counting

To ensure consistency, samples from single CFUs were used to inoculate 5 mLs of LB plus 50 µg/mL kanamycin. The cultures were then incubated at 37 °C and 180 rpm overnight. Next, 500 µL of culture was thoroughly mixed with the same volume of glycerol and the cells stored in a − 20 °C freezer.

For CFU counting, cells were grown to the exponential stage before cooling and diluting to ensure that the starting cell density was consistent in all screens. This involved adding 20 µL of the frozen cell stock to 30 mLs of LB broth plus 50 µg/mL kanamycin in a 250 mL conical flask. Cells were cultured at 37 °C and 200 rpm until the optical density at 600 nm (OD600) reached values between 0.3–0.4. Cells cultures were then centrifuged using a benchtop centrifuge at full speeds for 2 min. The cell pellet was resuspended in 1 mL of ice cold 0.1 M Tris pH 7.5, 100 mM NaCl. This wash step was repeated two more times to remove any traces of spent medium as well as to ensure cell division had stopped. Using the same ice cold buffer, each sample was then diluted to an OD600 of 0.2. This achieved a standardised average OD600 for 218 samples of 0.205 ± 0.015. 100 µL of this culture was used to plate on LB agar plates plus 50 µg/mL of kanamycin and 0–1 mM IPTG as indicated. Cells plated onto 0–0.1 mM IPTG were first diluted 10,000 times so that the average plate CFU count was between 200 and 400. Cells plated onto 0.2–1 mM were not diluted. To aid CFU phenotype counting, the brightness and contrast for each image was adjusted using GIMP [49] by approximately − 60 and + 50, respectively. All CFUs were counted by eye using the magnified versions of these images on a computer. Example plates are shown in Additional file 1: Fig. S2a while closeup pictures from the same plate images in Fig. 2b allow the identification of LW, LG and SCV phenotypes.

All samples taken were 1 mL but to stop additional cell growth and division before diluting and plating for CFU counting, the samples were immediately spun in a benchtop centrifuge at maximum speed for 2 min. After removing the spent medium, the cell pellet was placed on ice until further use. Before plating, cells were carefully resuspended using 1 mL of ice cold 0.1 M Tris, pH 7.5, 150 mM NaCl. Using a Nanodrop spectrophotometer the Resuspension OD600 was measured using the same buffer as a blank.

### CFU counts from over-expression of exogenous protein in liquid medium

Over-expression of exogenous proteins using the pET system is only ever carried out in liquid medium and not on agar plates. In addition, Fig. 2 data was generated by fixing the OD600 at 0.2 so that there was a consistent cell density that was tested against increasing IPTG concentrations. To understand what is happening in liquid medium during exogenous protein over-expression a new approach was implemented. The first part, the control, required counting the total number of CFUs from BL21(DE3) cells transformed with a pET expression vector without inducing exogenous protein over-expression over a normal timeframe for such an experiment (from 2–16 h). If the bactericidal effect of exogenous protein over-expression using IPTG concentrations > 0.1 mM holds true over all cell densities then the total CFU counts should decrease by > 99.9% (Fig. 4). To confirm this, uninduced samples were plated on LB agar containing 0.4 mM IPTG. Next, repeat exogenous protein over-expression trials were carried out but at an OD600 of ~ 0.6, protein induction was carried out with the addition of 0.4 mM IPTG. The cell density and IPTG concentration used for protein induction were based on those recommended by the manufacturer. Samples were plated on LB agar containing 0.4 mM IPTG. Thus, the cells were continually being exposed to IPTG both in liquid medium and on the plates. In this case, the number of CFUs would be expected to increase over time as the mutants begin to repopulate the system. Starter cultures of 5 mL LB, 50 µg/mL kanamycin plus 5 µL of the frozen stock was grown overnight at 37 °C and 235 rpm. These were used to inoculate sterile 50 mL of LB and 50 µg/mL kanamycin in a 250 mL flask. A pictorial representation of when samples were taken for CFU counting and analysis is illustrated in Additional file 1: Fig. S4. The cell culture’s OD600 was monitored until the OD600 value of approximately 0.6 was reached. At this stage, protein over-expression was started with the addition of 0.4 mM IPTG. After the indicated times, collected samples were treated as before to stop additional cell division. Next, depending on the total CFU numbers, samples were diluted up to 10 million times using ice cold buffer (0.1 M Tris, pH 7.5, 150 mM NaCl) before plating in duplicate on LB agar plus 50 µg/mL kanamycin and the indicated IPTG concentration of either 0 or 0.4 mM.

### Replica plating

Velveteen squares were sterilised by autoclaving before stretching tightly over a replica plating cylinder tool. A master plate containing cells grown on 0.75 mM IPTG was pressed on top of the replicating tool to transfer to the sterile velveteen square. Then a fresh LB agar plate with 0 mM IPTG was pressed on top of the tool. Replica plates were placed in an incubator at 37 °C for 48 h to provide enough time for potentially inhibited cells to grow.

### Genome sequencing

Selected mutant BL21-Gold(DE3)pLyS colonies or freshly transformed BL21(DE3) with the appropriate expression vector were grown overnight in 5 mL of LB with 50 µg/mL kanamycin at 37 °C and at 180 rpm. Cells from 1 mL aliquots were pelleted by centrifugation using a bench top centrifuge. Genomic DNA purification was achieved by using the components of a Nucleospin® plasmid DNA purification kit (Macherey–Nagel, catalogue number 740588) with a modified protocol. The cell pellet was resuspended using 250 µL of Resuspension buffer A1 containing RNAse A. Then, 250 µL of Lysis buffer was added and the tubes carefully inverted 5–6 times (taking care not to shear the DNA). The mixture was incubated at room temperature for 35 min to allow for the breakdown of RNA. Proteolytic cleavage of all proteins was next performed by adding 100 µg/mL of Proteinase K and incubating for 2.5 h at 55 °C. The sample was next added to a Nucleospin® DNA spin column and spun at 12,000 rpm for 1 min. Cleaning was performed by washing three times with 600 µL of Wash Buffer each time. The column was dried by 12,000 rpm centrifugation for 2 min. A total of 50 µL of de-ionised water was used to elute the DNA. If required, samples were concentrated using a rotatory evaporator at 45 °C.

### Bioinformatic analysis of genomic data

Whole genome sequencing was carried out using Illumina HiseqX at Novogene (Hong Kong) producing 150 bp paired-end reads. Raw fastqs were assessed for quality using fastQC (v0.11.3) and multiQC (v1.4). Reads were aligned to a combined fasta reference sequence containing the *E. coli* genome, and vector and insert sequences. Samples 7 and 8 were mapped against the reference genome ‘gi|296142109|gb|CP001509.3| *E. coli* BL21 (DE3), complete genome’, and samples 1–6 were mapped against the reference genome ‘gi|253322479|gb|CP001665.1| *E. coli* 'BL21-Gold(DE3)pLysS AG', complete genome’ both downloaded from NCBI genbank (https://www.ncbi.nlm.nih.gov/genbank/). The plasmid vector sequence ‘pET Biotin His6 GFP LIC cloning vector (H6-msfGFP)’ was available from Addgene (plasmid number 29725). The pLysS plasmid sequence was also downloaded from Addgene (plasmid number 3494).

Sequence reads were aligned using BWA (v0.7.17-r1188) and duplicates marked with Picard (v 2.15.0). Coverage was assessed using Samtools (v 1.6.0) and Bedtools (v2.26.0). Variants were called using GATK haplotype caller (v3.7) with ploidy set as 1 and annotated with SnpEff (v 4.3t). PanISA (v 0.1.4) was used to search for and identify insertion sequences using the ISFinder database. Alignment data were visualised with IGV (V2.3.98). Specifically, the promoters were visualised to identify erroneous mapping of reads between promoter sites, which allowed the conversion of the lacUV5 promoter’s-10 site (TATAAT) back to the lacI-10 sequence (TATGTT) to be determined.

## Supplementary Information

**Additional file 1: Figure S1. **The molecular control mechanism for exogenous protein production using the *E. coli* pET expression system. **Figure S2. **Representative plates of a 0.2 OD600 culture of BL21 (DE3) containing the sfGFP expression vector plated onto LB agar plus 50 µg/mL kanamycin and 0–1 mM IPTG. **Figure S3. **Over-expression of mdfA in BL21-Gold (DE3)pLysS is bactericidal. **Figure S4. **Methodology used to demonstrate the culture repopulation by mutant phenotypes after induction of exogenous protein over-expression. **Table S1. **Description of samples used for genome sequencing. **Table S2. **Observed genetic changes in comparison with published data. **Table S3. **Observed percentage of phenotypes from this data and previously published images. **Table S4. **Forward and reverse primers used to copy the *E. coli* K-12 MG1655 genes by PCR for ligation independent cloning into the H6msfGFP vector.

## Data Availability

All data generated or analysed during this study are included in this published article.
