# Supplementary material for: Protein over-expression in Escherichia coli triggers adaptation analogous to antimicrobial resistance
Source: Microb Cell Fact. 2021 Jan 11;20:13. doi: 10.1186/s12934-020-01462-6 (PMC7798265; doi:10.1186/s12934-020-01462-6)
Supplement: Supplementary file 1 — Additional file 1: Figure S1. The molecular control mechanism for exogenous protein production using the E. coli pET expression system. Figure S2. Representative plates of a 0.2 OD600 culture of BL21 (DE3) containing the sfGFP expression vector plated onto LB agar plus 50 µg/mL kanamycin and 0–1 mM IPTG. Figure S3. Over-expression of mdfA in BL21-Gold (DE3)pLysS is bactericidal. Figure S4. Methodology used to demonstrate the culture repopulation by mutant phenotypes after induction of exogenous protein over-expression. Table S1. Description of samples used for genome sequencing. Table S2. Observed genetic changes in comparison with published data. Table S3. Observed percentage of phenotypes from this data and previously published images. Table S4. Forward and reverse primers used to copy the E. coli K-12 MG1655 genes by PCR for ligation independent cloning into the H6msfGFP vector. [file 12934_2020_1462_MOESM1_ESM.docx]

**a**
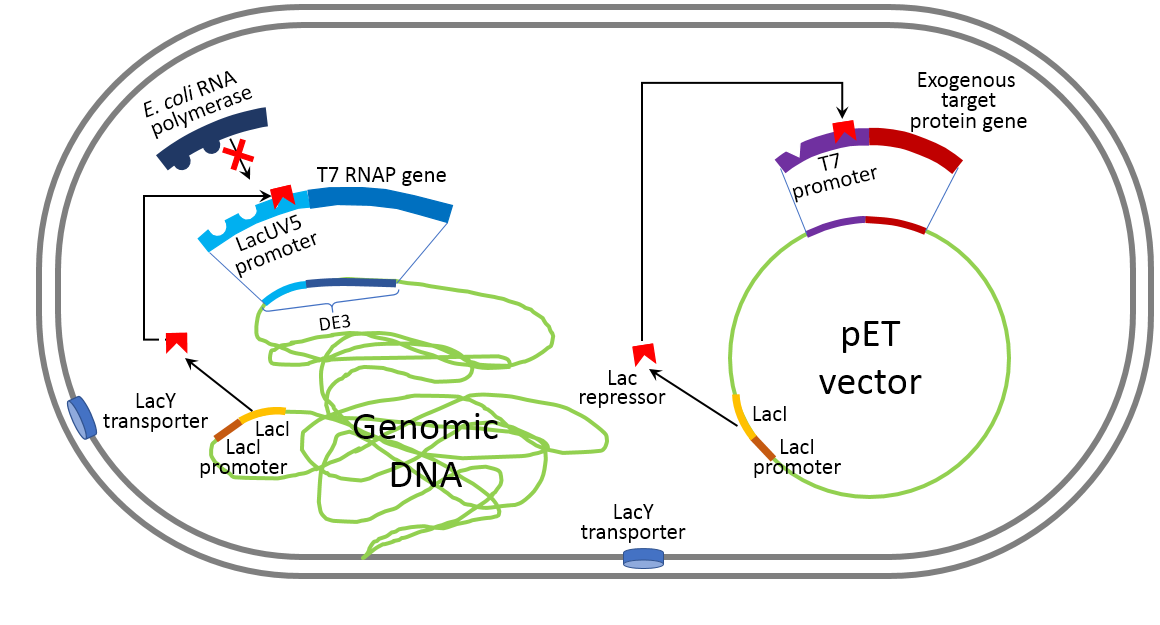


**b**


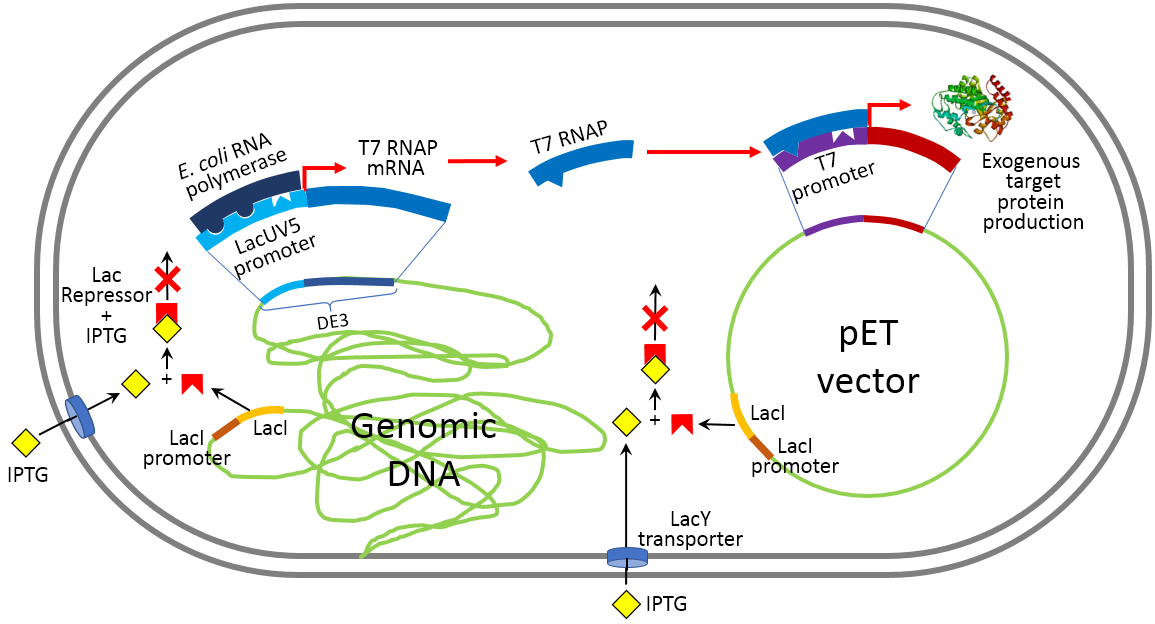


**Supplementary Figure 1. The molecular control mechanism for exogenous protein production using the *E. coli* pET expression system**. **a** The *E. coli* strains have the DE3 lysogen inserted into their genome and the lac repressor binds to the lacUV5 promoter preventing the transcription of the T7 RNAP polymerase (RNAP) gene. **b** Upon IPTG introduction, a complex is formed between the lac repressor and IPTG preventing it from binding to both the lacUV5 and T7 promoter sites. The endogenous *E. coli* RNA polymerase is then free to transcribe the T7 RNAP gene which, once converted to protein, recognises and binds to the T7 promoter site on the pET vector resulting in the production of the exogenous target protein. To date, the assumption has been made that this occurs in all DE3 transformed cells.

**a**


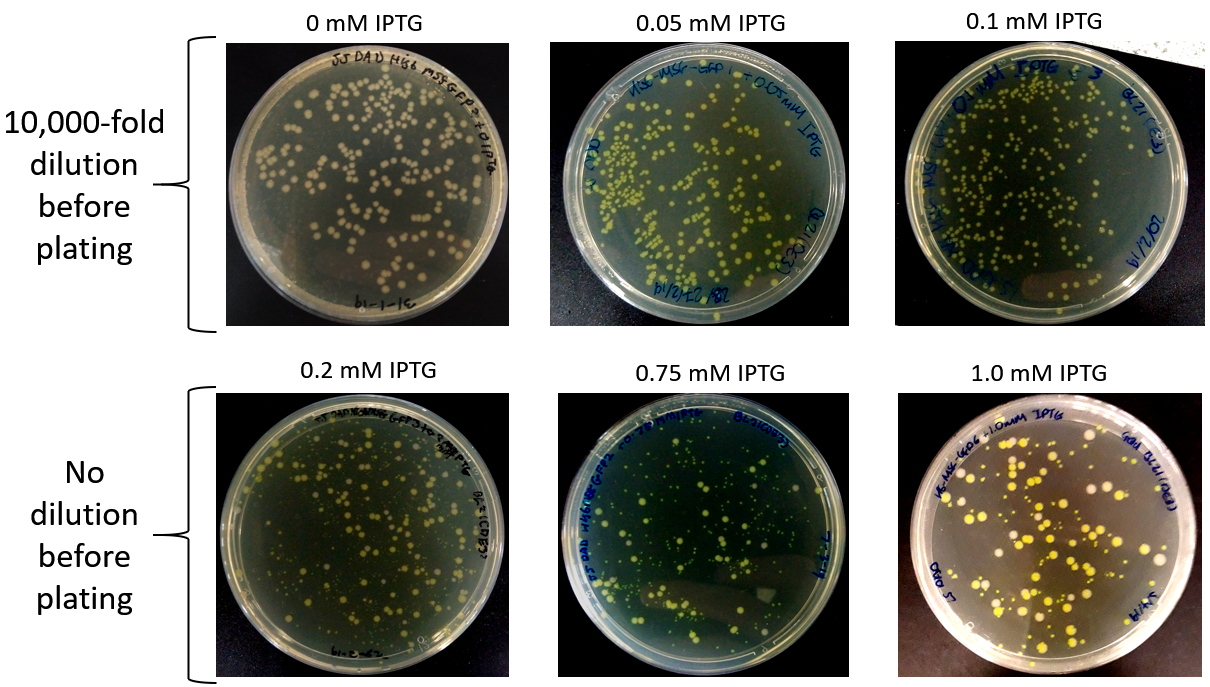


**b**


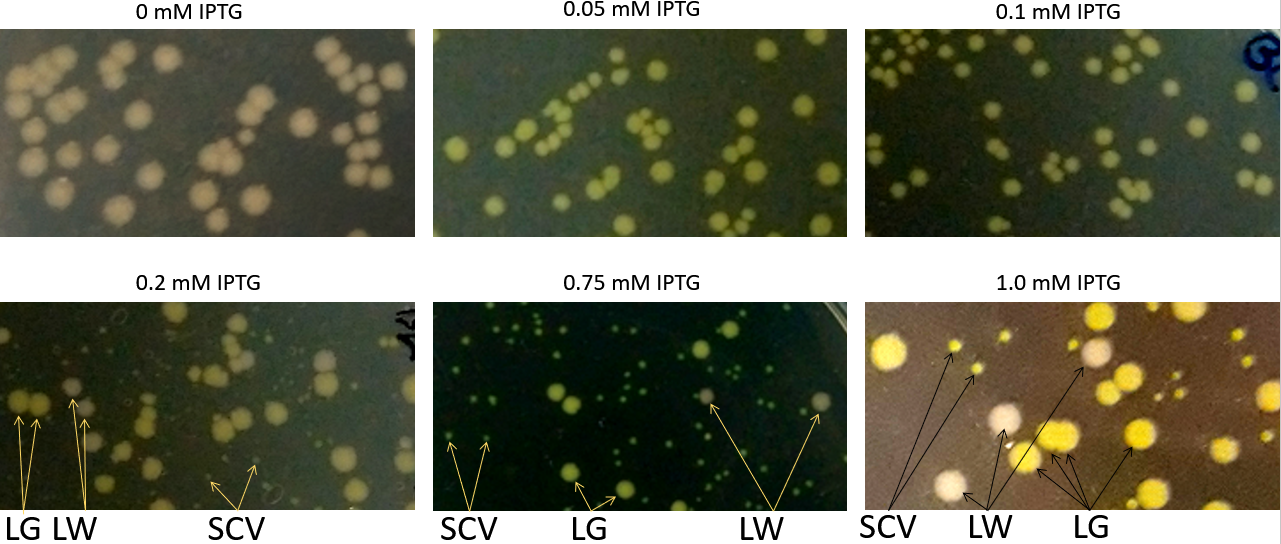


**Supplementary Figure 2. Representative plates of a 0.2 OD600 culture of BL21(DE3) containing the sfGFP expression vector plated onto LB agar plus 50 µg/mL kanamycin and 0 to 1 mM IPTG**. **a** is an overview of the plates showing the uniform CFU colours from the 0 to 0.1 mM IPTG screen while **b** is a close-up view of the (A) images to show the various CFUs and particularly the large white (LW), large green (LG) and small colony variant (SCV) that were only observed from the 0.2 – 1 mM plates.

**a b**


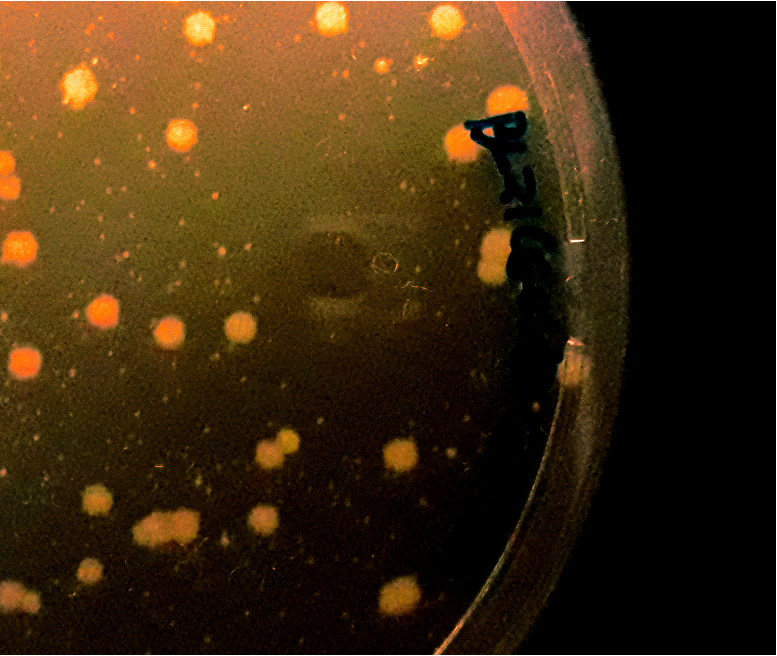

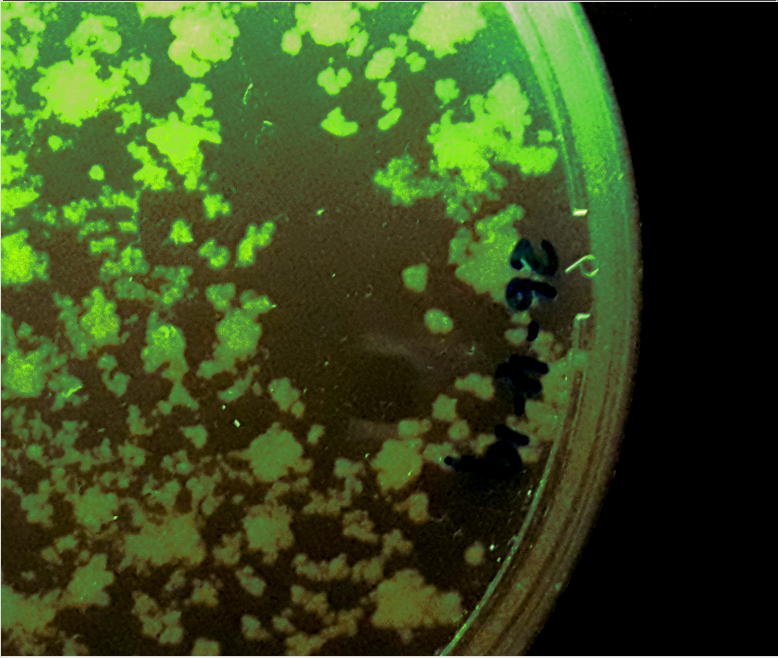


**c**


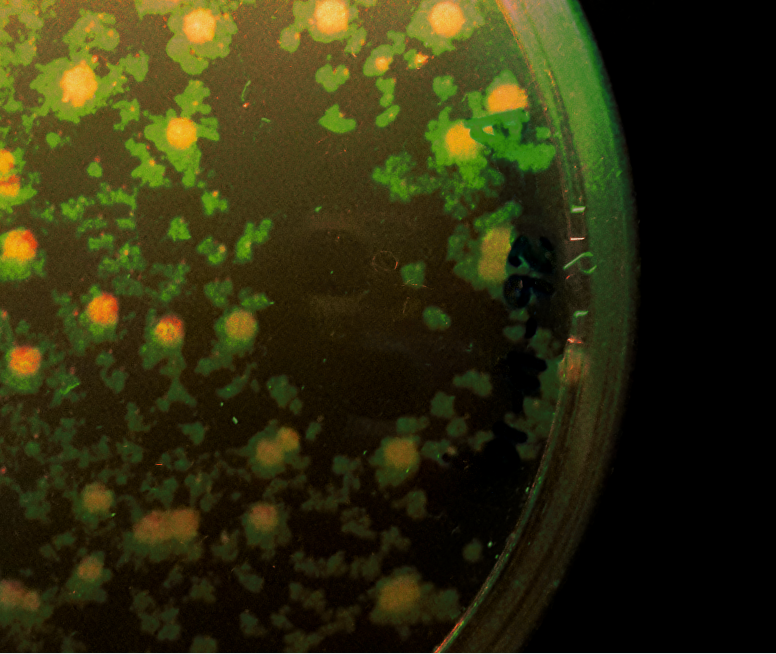


**Supplementary Figure 3. Over-expression of mdfA in BL21-Gold(DE3)pLysS is bactericidal**. **a** False colour showing large colonies and SCVs generated after incubation of ~70,000 cells on LB agar containing 0.75 mM IPTG. **b** False colour replica plate generated from A grown overnight on LB agar with 0 mM IPTG. **c** Manual overlap of A and B showing that only cells from pre-existing CFUs were able to grow on the 0 mM IPTG plate. False colouring and overlapping were performed using GIMP[49]. Approximately, 70,000 BL21-Gold(DE3)pLysS cells transformed with the mdfA expression vector were grown on LB agar containing 0.75 mM IPTG. This number of cells will produce a lawn on a standard sized LB agar culture plate. After overnight growth at 37 °C, replica plating onto LB agar plus kanamycin, but lacking IPTG, was performed. If exposure to high levels of IPTG is bacteriostatic then the high exogenous protein production rate, as a result of the use of 0.75 mM IPTG, would consume too many cellular resources, resulting first in the inhibition of cell growth and then cell death. Removal of the IPTG inducer by replica plating onto 0 mM IPTG would turn off exogenous protein production and thus be expected to restore cell growth therefore all cells would be expected to grow again, generating a bacterial lawn. If, however, the high induction rate of exogenous protein using 0.75 mM IPTG is bactericidal then only the modified surviving cells will grow on the 0 mM IPTG replica plate. Six such replica plates were generated, all of which showed the same results.

**a**


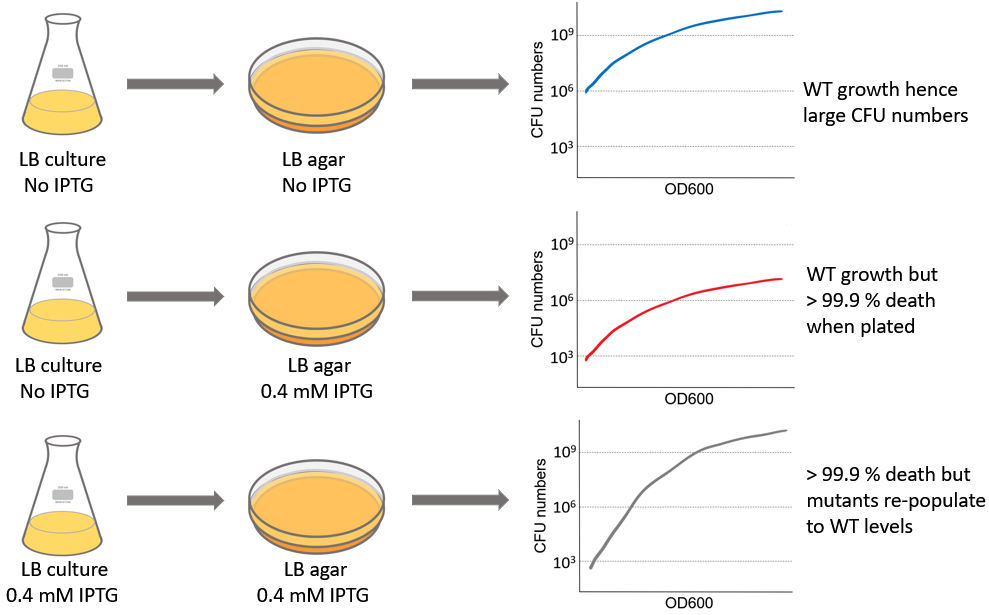


**b**


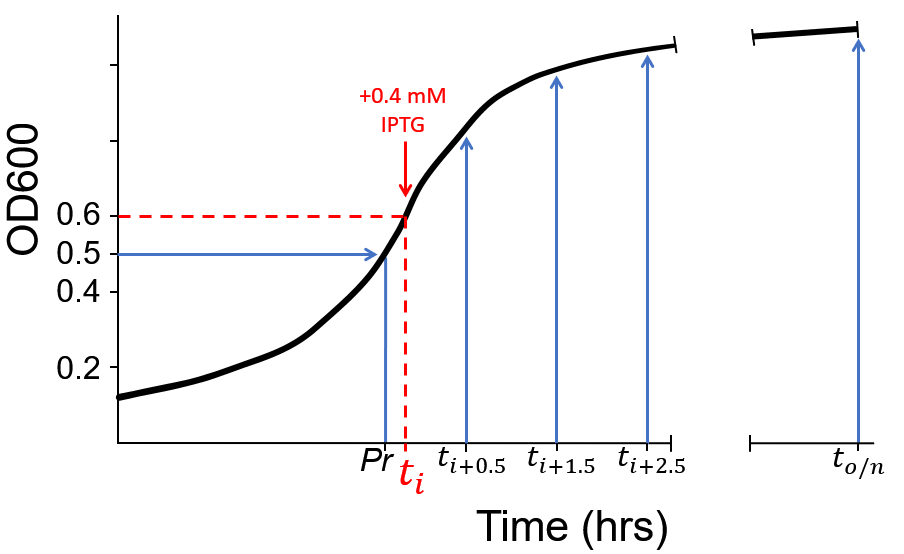


**Supplementary Figure 4. Methodology used to demonstrate the culture repopulation by mutant phenotypes after induction of exogeneous protein over-expression**. **a** To determine total CFU numbers samples from culture medium were plated on LB agar +/- IPTG as indicated. The three graphs are the expected changes in total CFU numbers against OD600. **B** Schematic growth curve indicating sampling times for CFU counting in order to determine change in phenotypes over time. Protein production was induced with the addition of 0.4 mM IPTG when the OD600 reached ~0.6. This time was noted as $t_{i}.$Preinduction (*Pr*) control samples were collected when the OD600 reached a value of 0.5. Additional samples were collected at times +0.5, +1.5, +2.5 hrs and an overnight (o/n) sample after the start of exogeneous protein induction ($t_{i}$).

**Supplementary Table 1. Description of samples used for genome sequencing.**

| Sample | Target protein expressed | SP  or IMP | IPTG exposure (mM) | Phenotype | Number of colonies in sample | Mutations |
| --- | --- | --- | --- | --- | --- | --- |
| 1 | sfGFP | SP | 1.0 | LW | 1 | IS10 insertion |
| 2 | sfGFP | SP | 1.0 | LG | 1 | lacUV5 > lacI promoter |
| 3 | mdfA | IMP | 1.0 | LW | 1 | IS10 insertion |
| 4 | mdfA | IMP | 1.0 | LG | 1 | lacUV5 > lacI promoter |
| 5 | sfGFP | SP | 0.01 | Bright green lawn | Many | 2x IS10 insertions in proportion of cells, and lacUV5 > lacI promoter in proportion of cells |
| 6 | mdfA | IMP | 0.01 | Pale green lawn | Many | None identified |
| 7 | sfGFP | SP | Never | n/a | 1 | WT BL21(DE3) |
| 8 | mdfA | IMP | Never | n/a | 1 | WT BL21(DE3) |

BL21-Gold(DE3)pLysS strain (Samples 1 – 6) was transformed with the appropriate target protein as indicated. Exogenous target expression levels are predicted based on the visibly observed intensity of green. Samples 7 and 8 are BL21(DE3) transformed with sfGFP and mdfA, respectively. Exogenous protein induction was never induced for samples 7 and 8. Samples 5 – 8 are controls. SP – soluble protein; IMP – integral membrane protein; LW – large white; LG – large green.

**Supplementary Table 2. Observed genetic changes in comparison with published data**

| **Reference** | **Basepair** | **Reference allele** | **Alternative allele** | **Sample** | | | | | | | | **Annotation** |
| --- | --- | --- | --- | --- | --- | --- | --- | --- | --- | --- | --- | --- |
|  |  |  |  | **1** | **2** | **3** | **4** | **5** | **6** | **7** | **8** |  |
| CP001509.3 | 453255 | G | A | - | - | - | - | - | - | 1 | 1 | missense_variant\|MODERATE\|acrB\|B21_00418\|transcript\|CAQ30935.1\|protein_coding\|1/1\|c.189C>T\|p.Gln63His |
| CP001509.3 | 1136454 | C | T | - | - | - | - | - | - | 1 | 1 | missense_variant\|MODERATE\|flgF\|B21_01081\|transcript\|CAQ31598.1\|protein_coding\|1/1\|c.92C>T\|p.Thr31Met |
| CP001509.3 | 2006172 | G | A | - | - | - | - | - | - | 1 | 1 | missense_variant\|MODERATE\|ybl97\|B21_01931\|transcript\|CAQ32448.1\|protein_coding\|1/1\|c.1007C>T\|p.Pro336Leu |
| CP001665.1 | 407916 | GT | G | 1 | 1 | 1 | 1 | 1 | 1 | - | - | upstream_gene_variant\|MODIFIER\|ECBD_0372\|ECBD_0372\|transcript\|ACT27443.1\|protein_coding\|\|c.-17delT |
| CP001665.1 | 692058 | C | T | . | . | . | . | . | 1 | - | - | *missense_variant\|MODERATE\|ECBD_R0013\|ECBD_R0013\|transcript\|ECBD_R0013-1.1\|protein_coding\|1/1\|c.67G>A\|p.Gly23Arg |
| CP001665.1 | 1502207 | T | TGCCTGATGCGACGCTGGCGCGTCTTATCAGGCCTACAAATCCGAGCCGTAGAGCCGTAGGCCGGATAAGGCGTTCACGCTGCATCCGGCACCCGGA | 1 | 1 | 1 | 1 | 1 | 1 | - | - | upstream_gene_variant\|MODIFIER\|ECBD_1422\|ECBD_1422\|transcript\|ACT28485.1\|protein_coding\|\|c.-2360_-2359insTCCGGGTGCCGGATGCAGCGTGAACGCCTTATCCGGCCTACGGCTCTACGGCTCGGATTTGTAGGCCTGATAAGACGCGCCAGCGTCGCATCAGGC |
| CP001665.1 | 2338226 | C | CA | 1 | 1 | 1 | 1 | 1 | 1 | - | - | upstream_gene_variant\|MODIFIER\|ECBD_2218\|ECBD_2218\|transcript\|ACT29249.1\|protein_coding\|\|c.-2376_-2375insT. Methyl-accepting chemotaxis sensory transducer |
| CP001665.1 | 2471531 | TC | T | 1 | 1 | 1 | 1 | 1 | 1 | - | - | upstream_gene_variant\|MODIFIER\|ECBD_2350\|ECBD_2350\|transcript\|ACT29377.1\|protein_coding\|\|c.-4852delG. Peptidase S49 domain protein |
| CP001665.1 | 2471557 | AC | A | 1 | 1 | 1 | 1 | 1 | 1 | - | - | upstream_gene_variant\|MODIFIER\|ECBD_2350\|ECBD_2350\|transcript\|ACT29377.1\|protein_coding\|\|c.-4878delG. Peptidase S49 domain protein |
| CP001665.1 | 2471565 | CG | C | 1 | 1 | 1 | 1 | 1 | 1 | - | - | upstream_gene_variant\|MODIFIER\|ECBD_2350\|ECBD_2350\|transcript\|ACT29377.1\|protein_coding\|\|c.-4886delC. Peptidase S49 domain protein |
| CP001665.1 | 3125367 | G | GA | 1 | 1 | 1 | 1 | 1 | 1 | - | - | upstream_gene_variant\|MODIFIER\|ECBD_R0060\|ECBD_R0060\|transcript\|ECBD_R0060-1.1\|protein_coding\|\|c.-11_-10insA |
| CP001665.1 | 3125513 | A | T | . | . | . | . | . | 1 | - | - | *Stop_gained\|HIGH\|tRNA\|EBG00001118646\|transcript\|EBT00001708496.1\|protein_coding\|1/1\|c.32T>A\|p.Leu11* |
| CP001665.1 | 3809720 | C | CGAACGCCTTATCCGGCCTACGGTTCGGCACAAACCTGTAGGCATGATAAGACGCGACAAGCGTCGCATCAGGCATTATGCGCCGACTGCCGGATGCGGCGTGAACGCCTTATCCGGCCTACGGTTCGGCACAGACTTGTAGGCATGATAAGACGCGACAAGCGTCGCATCAGGCATTGTGCGCCAACTGCCGGATGCGGCGT,T | 1 | 1 | 1 | 2 | 1 | 1 | - | - | upstream_gene_variant\|MODIFIER\|ECBD_3638\|ECBD_3638\|transcript\|ACT30634.1\|protein_coding\|\|c.-3649G>A. Thymidine phosphorylase deoA. |
| CP001665.1 | 4373557 | T | G | 1 | 1 | 1 | 1 | 1 | 1 | - | - | upstream_gene_variant\|MODIFIER\|ECBD_4153\|ECBD_4153\|transcript\|ACT31136.1\|protein_coding\|\|c.-30A>C. Sugar phosphate permease-like protein araJ – called yihN. |
| H6-msfGFP | 2320 | ATAT | A | 1 | 1 | 1 | 1 | 1 | 1 | 1 | 1 | bom |
| H6-msfGFP | 3890 | G | A | 1 | 1 | 1 | 1 | 1 | 1 | 1 | 1 | lacI |
| pLysS | 944 | T | TC | 1 | 1 | 1 | 1 | 1 | 1 | - | - | p15A ori |
| pLysS | 1631 | G | T | 1 | 0 | 0 | 0 | 0 | 0 | - | - | tet |
| pLysS | 1636 | A | ACAATTGAC,AAAATTGAC | 1 | 0 | 2 | . | . | 0 | - | - | tet |
| pLysS | 1638 | G | A | 1 | . | . | . | . | . | - | - | tet |
| pLysS | 3909 | GT | G | 1 | 1 | 1 | 1 | 1 | 1 | - | - | N/A |
| pLysS | 4406 | C | CA | 1 | 1 | 1 | 1 | 1 | 1 | - | - | downstream of camR |
| pLysS | 4521 | G | A | 1 | 1 | 1 | 1 | 1 | 1 | - | - | camR |

Variants called using GATK (with ploidy=1). Reads mapped to either "gi|253322479|gb|CP001665.1| E. coli 'BL21-Gold(DE3)pLysS AG" (samples 1 – 6) or "gi|296142109|gb|CP001509.3| E. coli BL21(DE3), complete genome" (samples 7 & 8) as well as the vectors "pET Biotin His6 GFP LIC cloning vector (H6-msfGFP)" and "pLysS". Annotation provided by SnpEff for the genome sequences and https://www.addgene.org/ for the vectors. (Reference not present in sample "-", variant not called due to lack of coverage "."). Under Annotation, those starting with a * appears to be an issue with reads from the plasmid mapping to the genome in a tRNA region.

**Supplementary** **Table 3. Observed percentage of phenotypes from this data and previously published images**

| **STRAIN** | **Vector** | **IPTG (mM)** | **Marker** | **LW** | **LG** | **L total** | **SCV** |
| --- | --- | --- | --- | --- | --- | --- | --- |
| *BL21(DE3) | H6msfGFP | 0.2-1.0 | Kanamycin | 3.3 | 20.8 | 24.1 | 75.9 |
| *BL21-Gold(DE3)pLysS | H6msfGFP | 0.2-1.0 | Kanamycin | 15.7 | 27.2 | 42.9 | 57.1 |
| BL21(DE3)  Miroux & Walker, 1996[12] | pMW7 | 0.7 | Ampicillin | n/a | | 30.6 | 69.4 |
| BL21(DE3)  Hattab et al, 2014[11] | pT7GFP | 1.0 | Ampicillin | n/a | | 52.3 | 47.7 |
| BL21(DE3)  Schlegel et al, 2015[15] | pMV7-GFP-Xa | 0.7 | Ampicillin | n/a | | 34.2 | 65.8 |

Large white (LW), large green (LG), LW + LG = large total (L total) and small colony variant (SCV) phenotypes. Marked with a star are the data calculated from this work which is the percentage average over all 0.2 – 1 mM IPTG plates. The remaining percentages were calculated from a single image of a LB agar plate from the published data as indicated. For this data, due to differences in data acquisition, it was not possible to distinguish LW and LG hence a combined total (L total) was used instead. The final IPTG concentration for each experiment is indicated as well as the vector and the selective antibiotic marker used in the experiments.

**Supplementary Table 4. Forward and reverse primers used to copy the *E. coli* K-12 MG1655 genes by PCR for ligation independent cloning into the H6msfGFP vector.**

| Forward Primers | |
| --- | --- |
| **Name** | **Sequence** |
| mdfA-f1 | TACTTCCAATCCAATGCACAAAATAAATTAGCTTCCGGT |
| mdtG-f1 | TACTTCCAATCCAATGCAGACACCCCTATAAACTGGAAA |
| amtB-f1 | TACTTCCAATCCAATGCAAAGATAGCGACGATAAAAACT |
| bcr-f1 | TACTTCCAATCCAATGCATCGTCGTTTGCTATTGTTTTT |
| setB-f1 | TACTTCCAATCCAATGCAAGCGCGAAATCGTTTGACCTG |
| mdtL-f1 | TACTTCCAATCCAATGCATCCCGCTTTTTGATTTGTAGT |
| Reverse Primers | |
| **Name** | **Sequence** |
| mdfA-r1 | CTCCCACTACCAATGCCCATCTGTTTATCTTTTAAAAA |
| mdtG-r1 | CTCCCACTACCAATGCCGGGTATTCGACGACGACGTAG |
| amtB-r1 | CTCCCACTACCAATGCCCGCGTTATAGGCATTCTCGCC |
| bcr-r1 | CTCCCACTACCAATGCCCCGTTTTTTCGGCCGACTGGC |
| setB-r1 | CTCCCACTACCAATGCCAACATCTTTAATCCGCAGTAA |
| mdtL-r1 | CTCCCACTACCAATGCCCGCGACGAACATAATCAGCAA |
